# Treatment approach to coronary aneurysms: percutaneous or surgical?

**DOI:** 10.1186/s13019-020-01185-0

**Published:** 2020-06-09

**Authors:** Ahmet Güner, Ezgi Gültekin Güner, Ali Kemal Kalkan, Fatih Uzun, Mehmet Ertürk

**Affiliations:** Department of Cardiology, Mehmet Akif Ersoy Thoracic and Cardiovascular Surgery Training and Research Hospital, 34303 Kucukcekmece, Istanbul, Turkey

**Keywords:** Giant, Coronary arteries, Aneursym

## Abstract

We have recently read with great interest the article by Teng P et al. entitled “Giant right coronary artery aneurysm mimicking a right intra-ventricular mass: a case report”. We appreciate the authors for their reports describing the treatment of a giant right coronary aneurysm mimicking a right intra-ventricular mass. On the other hand, we believe that there are some major drawbacks that need to be addressed.

## Main text

To the Editor,

We have recently read with great interest the article by Teng P et al. entitled “Giant right coronary artery aneurysm mimicking a right intra-ventricular mass: a case report” [1]. We appreciate the authors for their reports describing the treatment of a giant right coronary aneurysm mimicking a right intra-ventricular mass. On the other hand, we believe that there are some major drawbacks that need to be addressed.

First, giant coronary artery aneurysms are rare clinical entities that may have catastrophic consequences such as myocardial infarction [2]. Angiographic incidence of giant coronary artery aneurysms has been reported to be 0.02–0.2% and it is more common in men and mostly seen in right coronary artery. The common causes of an aneurysm in adults are atherosclerosis, vasculitis (such as Takayasu and Behçet) whereas in children is associated with Kawasaki’s disease [3]. In this case report, the authors stated that the patient did not have kawasaki disease, but was the patient evaluated for connective tissue disease, vasculitis and especially for Behçet? We know that the coexistence of vasculitis and giant coronary aneurysms is not as rare as it seems [4].

Second, in the last 10 years, case reports on percutaneous treatment of giant coronary aneurysms have been reported in the literature [5, 6]. It should be considered especially if the proximal and distal edge of the aneurysm is suitable for percutaneous stent implantation. In this case report, was the patient evaluated for percutaneous treatment before surgery? We know that the aneurysm can be treated successfully if the aneurysmal formation with good distal vascular bed is suitable for stent implantation of the distal and proximal vascular bed.

## Data Availability

Not applicable.
